# SUMO: Glue or Solvent for Phase-Separated Ribonucleoprotein Complexes and Molecular Condensates?

**DOI:** 10.3389/fmolb.2021.673038

**Published:** 2021-05-07

**Authors:** Jan Keiten-Schmitz, Linda Röder, Eran Hornstein, Michaela Müller-McNicoll, Stefan Müller

**Affiliations:** ^1^Faculty of Medicine, Institute of Biochemistry II, Goethe University, Frankfurt, Germany; ^2^Department of Molecular Genetics, Weizmann Institute of Science, Rehovot, Israel; ^3^Department of Molecular Neuroscience, Weizmann Institute of Science, Rehovot, Israel; ^4^Faculty of Biosciences, Institute for Molecular Biosciences, Goethe University, Frankfurt am Main, Germany

**Keywords:** SUMO, RNF4, PML, membrane-less organelles, nucleolus, stress granules, splicing

## Abstract

Spatial organization of cellular processes in membranous or membrane-less organelles (MLOs, alias molecular condensates) is a key concept for compartmentalizing biochemical pathways. Prime examples of MLOs are the nucleolus, PML nuclear bodies, nuclear splicing speckles or cytosolic stress granules. They all represent distinct sub-cellular structures typically enriched in intrinsically disordered proteins and/or RNA and are formed in a process driven by liquid-liquid phase separation. Several MLOs are critically involved in proteostasis and their formation, disassembly and composition are highly sensitive to proteotoxic insults. Changes in the dynamics of MLOs are a major driver of cell dysfunction and disease. There is growing evidence that post-translational modifications are critically involved in controlling the dynamics and composition of MLOs and recent evidence supports an important role of the ubiquitin-like SUMO system in regulating both the assembly and disassembly of these structures. Here we will review our current understanding of SUMO function in MLO dynamics under both normal and pathological conditions.

## Introduction

Most cellular processes are compartmentalized in membranous or membrane-less organelles (MLOs, also termed molecular condensates). Prototypical MLOs in the nucleus are the nucleolus, paraspeckles, nuclear speckles (NS), Cajal bodies, PML nuclear bodies (PML NBs) or nuclear stress bodies (nSBs), and in the cytoplasm P-bodies and stress granules (SGs) (Banani et al., 2017; Alberti and Hyman, 2021). All these structures typically contain disordered proteins and/or RNA and form in a process that is driven by liquid-liquid phase separation (LLPS). LLPS describes the condensation of biological macromolecules in a dense phase that resembles liquid droplets and is stabilized by multivalent interactions. Intrinsically disordered regions (IDRs) of RNA binding proteins (RBPs) often play important roles in these condensates, in which specific RNAs or proteins act as scaffolds that recruit other client proteins. Several MLOs function as RNA or protein quality control centers and, accordingly, their formation, disassembly and composition are highly sensitive to cellular stress, including proteotoxic stress (Gartner and Muller, 2014; Advani and Ivanov, 2019). Post-translational modifications (PTMs) have emerged as regulators of phase separation in the dynamics of MLOs and accumulating evidence points to the involvement of the SUMO system in these processes (Banani et al., 2016; Hofweber and Dormann, 2019). The SUMO pathway constitutes an evolutionary conserved ubiquitin-like post-translational modification system. SUMO (Small ubiquitin-related modifier) proteins (SUMO1,2,3 in humans) are covalently attached to a multitude of cellular proteins via lysine-linked isopeptide bonds (Flotho and Melchior, 2013; Cappadocia and Lima, 2018). At the amino acid level human SUMO2 and SUMO3 are 98% identical to each other and share about 50% identity to SUMO1. Conjugation of all three modifiers involves the heterodimeric E1 enzyme (AOS1/UBA2), the E2 enzyme UBC9 and a relatively small set of E3 SUMO ligases serving as specificity factors. SUMOylation is reversed by SUMO-specific isopeptidases. Notably, compared to the ubiquitin (Ub) system, the SUMO conjugation-deconjugation machinery is far less complex and SUMO E3 ligases or isopeptidases mostly target groups of related proteins that are physically and functionally connected (Jentsch and Psakhye, 2013). SUMO can be conjugated as a monomer, but also forms different types of polymeric chains via internal lysine residues (Keiten-Schmitz et al., 2019; Perez Berrocal et al., 2019). Compared to SUMO2/3, SUMO1 is less prone to chain formation and at least in some instances terminates SUMO2/3 chains (Jansen and Vertegaal, 2021). SUMOylation generally coordinates the plasticity of protein networks by modulating protein-protein interactions. This is mediated by specific SUMO interaction motifs (SIMs) that bind to SUMO conjugates thereby reading and interpreting the SUMO signal. There are multiple examples, where SUMO-SIM interactions can function in a “glue-like” manner to control the assembly of protein complexes (Matunis et al., 2006). SUMO chains, however, can trigger a particular signaling cascade, known as the SUMO-targeted ubiquitin ligase (StUbL) pathway (Kumar and Sabapathy, 2019). In this pathway, polySUMOylated proteins are bound by distinct ubiquitin ligases that harbor tandemly repeated SIMs. In mammals the RING-type E3 ligases RNF4 and RNF111 function as StUbLs triggering proteolytic or non-proteolytic ubiquitylation of polySUMOylated proteins, thereby directly bridging SUMO signaling to the Ub machinery. SUMO chain formation and the StUbL pathway are induced in response to proteotoxic or genotoxic stress (Jansen and Vertegaal, 2021). Under proteotoxic stress SUMO-primed ubiquitylation by RNF4 contributes to protein quality control by degrading misfolded nuclear proteins (Gartner and Muller, 2014; Guo et al., 2014). In the genotoxic stress response StUbLs are critical for remodeling of protein complexes (Keiten-Schmitz et al., 2019). The importance of the StUbL pathway for resolving protein complexes is best exemplified in the DNA damage response, where the disassembly of DNA repair complexes at sites of DNA damage is often mediated by polySUMO-primed RNF4-mediated ubiquitylation either triggering their degradation or their extraction from chromatin (Keiten-Schmitz et al., 2019). The latter process typically involves the AAA-ATPase p97/VCP and its co-factors (Bergink et al., 2013). In the following sections we will exemplify the role of the SUMO system in controlling the dynamics of membrane-less organelles.

## SUMO and the Dynamics of PML Nuclear Bodies

**FIGURE 1:**
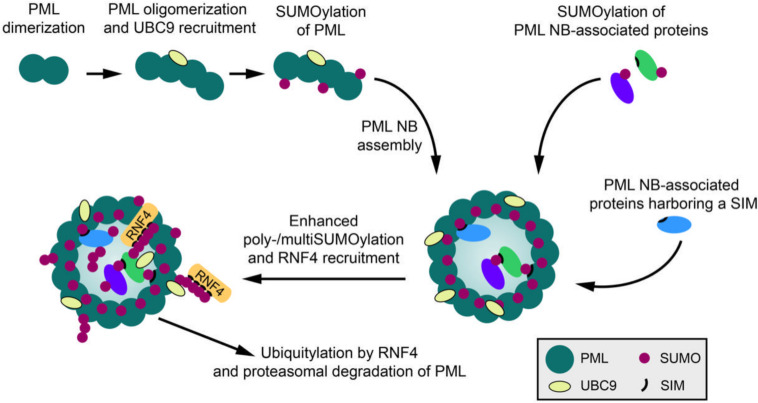
SUMO and the dynamics of PML nuclear bodies. PML NB formation begins with PML oligomerization via its N-terminal domain. UBC9 is recruited to PML oligomers and PML is SUMOylated. PML SUMOylation and consequential SUMO-SIM interactions further promote PML NB assembly and the recruitment of PML NB-associated proteins into PML NBs. Stimuli such as arsenic trioxide treatment can lead to multi- and polySUMOylation of PML and NB-associated proteins. The StUbL RNF4 can subsequently ubiquitylate PML, thereby targeting it for proteolytic degradation and causing the clearance of PML NBs.

A paradigm for SUMO-SIM-dependent complex assembly and phase separation are PML (promyelocytic leukemia protein) nuclear bodies. The biomedical interest in PML NBs stems from the initial observation that the structural integrity of these macromolecular assemblies is lost in acute promyelocytic leukemia (APL). Disruption of NBs in APL is caused by expression of the oncogenic fusion protein PML-RARα (PML- retinoic acid receptor alpha) resulting from the aberrant t(15, 17) chromosomal translocation (Lallemand-Breitenbach and de The, 2018). PML NB biology is still not fully understood, but one well-established role is their function as hubs for post-translational modifications and centers of nuclear protein quality control (Gartner and Muller, 2014; Guo et al., 2014; Sha et al., 2019). Newly synthesized aberrant polypeptide chains, such as defective ribosomal products (DRiPs), or misfolded proteins, e.g., polyQ proteins, are sequestered into PML NBs, where they are cleared by the chaperone machinery or the ubiquitin proteasome system (Guo et al., 2014; Mediani et al., 2019a,b; Sha et al., 2019). It has been proposed that PML itself can recognize aberrant or misfolded proteins subsequently triggering their SUMOylation and StUbL-mediated ubiquitylation (Gartner and Muller, 2014; Guo et al., 2014). A role of PML NBs as centers of proteostasis is further supported by their enhanced formation in response to reactive oxygen species suggesting that they act as sensors for oxidative stress (Jeanne et al., 2010; Sahin et al., 2014). PML, which functions as the scaffold and organizer of this multiprotein complex, is expressed in seven different isoforms in humans and belongs to the tripartite motif (TRIM) family of proteins, characterized by a RING finger domain, two B-box zinc finger domains and a coiled-coil region (Jensen et al., 2001). PML represents a major cellular target for covalent modification by SUMO and also harbors a SIM for non-covalent SUMO binding (Muller et al., 1998; Zhong et al., 2000; Shen et al., 2006). Similarly, most proteins associated with PML NBs are modified by SUMO and/or contain SIMs. A plethora of cell-biological studies over more than 20 years led to a model, in which SUMO-SIM interactions provide the glue for the assembly of mature PML NBs. The biogenesis of PML NBs occurs in at least two steps (Figure 1). The initial nucleation phase, which generates an outer shell primarily comprised of PML, requires oligomerization of PML. It has been proposed that disulfide bridges between oxidized PML monomers as well as intermolecular non-covalent interactions between its RBCC domains are the major drivers of this event (Jeanne et al., 2010; Sahin et al., 2014). At least for some PML isoforms SUMO-SIM-dependent oligomerization also contributes to this process (Zhong et al., 2000; Shen et al., 2006; Li et al., 2017). The subsequent maturation phase of PML NBs is triggered by the recruitment of multiple proteins to the inner core of the scaffold. Importantly, this process is primarily dictated by SUMO-SIM-dependent protein-protein interactions. It is indeed well established that SUMOylation of PML induces recruitment of other SIM-containing factors to these bodies, such as DAXX, HIPK2 or SP100 (Figure 1; Weidtkamp-Peters et al., 2008; Sung et al., 2011). Upon recruitment to NBs these factors typically also undergo covalent modification by SUMO, amplifying the assembly process. More recent *in vitro* biochemical and biophysical studies strengthened this conceptual framework and provided evidence that phase separation in PML NBs is driven by SUMO polymers that recruit SIM-containing proteins (Banani et al., 2017). In line with the current model, partitioning of these clients into PML NBs requires SUMO-SIM binding and depends on the levels of PML SUMOylation. By controlling SUMO conjugation-deconjugation, cells can regulate PML NB composition. This is exemplified by an increase in their number and size upon inactivation of the SUMO deconjugase SENP6 (Mukhopadhyay et al., 2006; Hattersley et al., 2011), which limits chain formation on PML. Another way to control PML dynamics is the regulation of SUMO-SIM interactions through additional PTMs in either SUMO or the SIM region (Stehmeier and Muller, 2009; Ullmann et al., 2012; Cappadocia et al., 2015; Cappadocia and Lima, 2018). Notably, SUMO-SIM-dependent LLPS also contributes to the formation of ALT (alternative lengthening of telomeres)-associated PML NBs that mediate telomerase-independent telomere maintenance in a subset of cancer cells (Min et al., 2019; Zhang et al., 2020). However, one important aspect of the SUMO-SIM glue model in PML NB condensation is that polymeric SUMO chains on PML can also recruit and activate the StUbL RNF4, ultimately leading to the proteolytic degradation of PML and the disassembly of the NBs (Lallemand-Breitenbach et al., 2008; Tatham et al., 2008; Figure 1). This scenario can be experimentally induced by treating cells with arsenic trioxide, which triggers polySUMOylation of PML. Initially this causes an increase in size and number of PML NBs via recruitment of SIM-containing clients, but at a later stage leads to the complete disappearance of NBs. SUMO-dependent degradation of PML, followed by disassembly of NBs, is also observed upon infection with Herpes simplex virus, which encodes the viral StUbL ICP0 (Muller and Dejean, 1999; Jan Fada et al., 2020). Altogether these data demonstrate that, dependent on the nature of the SUMO signal, SUMOylation can exert dual functions on MLOs by either fostering their assembly or disassembly. PolySUMO chains on PML or other NB component that exceed a certain length are preferentially targeted by RNF4-mediated ubiquitylation and proteasomal degradation. In this context, SUMO loses its glue-like functions and contributes to the dissolution of MLOs by mediating scaffold degradation. Notably, recent work suggests that at least in certain cases stress-induced SUMO conjugation can keep unfolded proteins soluble and prevent their accumulation into insoluble aggregates independent from ubiquitylation (Liebelt and Vertegaal, 2016; Liebelt et al., 2019).

## SUMO Conjugation-Deconjugation in the Nucleolus

The nucleolus is another prototypic membrane-less organelle forming by liquid-liquid phase separation (Lafontaine et al., 2020). Nucleoli are complex structures, where ribosomal and non-ribosomal proteins form a macromolecular network through interactions with RNA, such as rRNAs or snoRNAs. Nucleoli are organized in three morphologically distinct sub-regions, where successive steps of ribosome biogenesis take place. The inner core, termed fibrillar center (FC), is the site of rRNA transcription. Early and late nucleolar maturation of ribosomal subunits occur in the dense fibrillar component (DFC) and in the more peripheral granular components (GC), respectively. FC, DFC, and GC likely represent coexisting, immiscible, liquid phases determined by differences in the biophysical properties of their constituents. A key organizer of the liquid-like structure of the GC is nucleophosmin (NPM1 or B23). It has been proposed that multiple NPM1-regulated LLPS mechanisms influence the ordered assembly of pre-ribosomal particles and their exit from the nucleolus (Mitrea et al., 2018). Importantly, there is evidence that the function of NPM1 is interconnected with the SUMO system by stabilization of the SUMO deconjugases SENP3 and SENP5 and by recruiting them to the GC region (Yun et al., 2008; Raman et al., 2014). SENP3/5 control the SUMOylation status of many nucleolar, ribosomal and non-ribosomal proteins and the lack of nucleolar SENP3 induces unscheduled SUMOylation at 60S pre-ribosomes leading to nucleolar exit of immature pre-60S particles (Finkbeiner et al., 2011; Castle et al., 2012; Raman et al., 2016). Noteworthy, NPM1 itself is a major nucleolar target of SUMOylation, which inhibits 28S maturation (Haindl et al., 2008). Although it remains to be determined whether the balance of SUMO conjugation-deconjugation on NPM1 or other nucleolar proteins affects the different LLPS processes in the nucleolus, it is attractive to speculate that SUMO may modulate protein-protein or RNA-protein interactions that drive phase separation. In support of this idea, SUMOylation of the snoRNP component NOP58 was shown to facilitate its interaction with Box C/D snoRNA, thereby targeting snoRNPs to the nucleolus (Westman et al., 2010). Similarly, miscibility of Dyskerin (DKC1) within the nucleolar DFC was proposed to rely on SUMO-dependent binding of DKC1 to a SIM in GAR1, a component of the H/ACA snoRNP complex (MacNeil et al., 2021). Altogether, these data suggest that SUMO may contribute to phase separation in the nucleolar compartment. Notably, under specific conditions the StUbL pathway also plays a role in resolving nucleolar condensates as exemplified by the SUMO/RNF4-dependent nucleolar release of repair complexes that act on damaged rDNA in the nucleolus (Capella et al., 2021).

Importantly, new data indicate that in addition to their crucial role in ribosome biogenesis nucleoli exert critical functions in protein quality control and proteostasis (Alberti and Carra, 2019; Amer-Sarsour and Ashkenazi, 2019; Mende and Muller, 2021). Similar to what was observed in PML NBs, aberrant translation products or misfolded proteins accumulate transiently in nucleoli for further clearance by the chaperone machinery (Frottin et al., 2019; Mediani et al., 2019b). Intriguingly, under stress conditions, misfolded proteins enter the GC region, where association with NPM1 or other GC components prevent their irreversible aggregation. Considering that NPM1 SUMOylation is strongly induced upon proteotoxic stress and that at least *in vitro* SUMOylation functions as a general solubility “tag” it is tempting to speculate that SUMO may contribute to this process.

## SUMO Control of the Splicing Machinery and Nuclear Speckles

Nuclear speckles are phase-separated MLOs with key functions in mRNA processing and quality control (Galganski et al., 2017). Acting as a physical barrier, they temporarily retain incompletely processed and export-incompetent mRNA-protein complexes (mRNPs) after their release from chromatin (Girard et al., 2012). Nuclear speckles also retain and release mRNPs as part of a regulated, nuclear stress response (Hochberg-Laufer et al., 2019). Furthermore, it was recently proposed that the interface of phase-separated and non-phase-separated areas of nuclear speckles spatially organize the biochemical reaction of alternative splicing (Liao and Regev, 2021). Pre-mRNA splicing is catalyzed by the spliceosome that assembles at each intron from five small nuclear ribonucleoprotein particles, termed U1, U2, U4, U5, and U6 snRNP. Each snRNP consists of a small nuclear RNA (snRNAs) and a large set of associated proteins (Wahl et al., 2009). Assembly of spliceosomes starts with the formation of the A complex comprising U1 and U2 snRNP bound to the intron. Binding of the U4/U6U5 tri-snRNP generates the B complex, which is converted to its active form releasing U1 and U4 snRNP. The C complex then catalyzes intron excision and ligation of the exons followed by spliceosome disassembly (Kastner et al., 2019).

**FIGURE 2:**
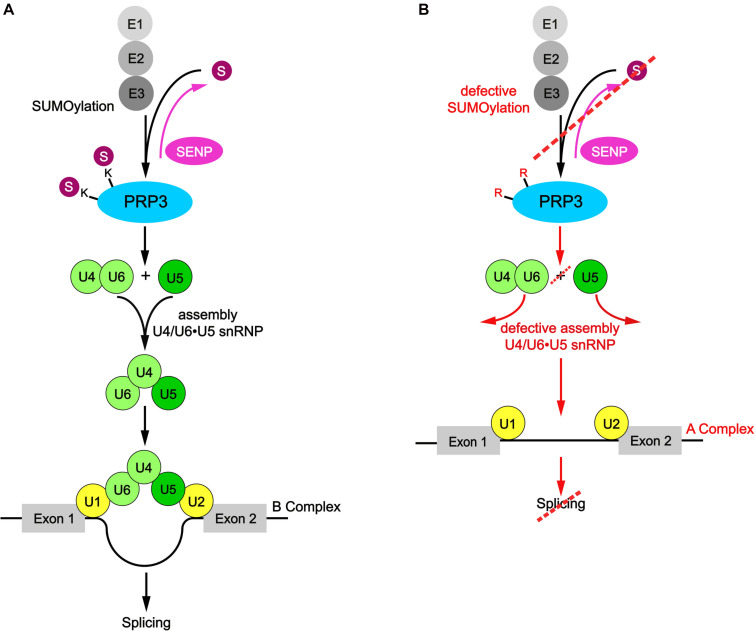
SUMOylation of PRP3 promotes U4/U6U5 tri-snRNP formation. **(A)** The human PRP3 protein, as a component of the U4/U6 di-snRNP, is a SUMOylation target and promotes U4/U6U5 tri-snRNP formation to convert the A complex into the active B complex by interacting with U2 and U5 thereby promoting the splicing process. **(B)** After mutation of the relevant lysine residues into arginine, the SUMO-deficient PRP3 fails to co-precipitate U2 and U5 snRNAs resulting in hampered U4/U6U5 tri-snRNP assembly and shows diminished recruitment to splice sites indicating that SUMOylation of PRP3 promotes U4/U6U5 tri-snRNP formation.

Nuclear speckles are built from two RBPs, SRRM2, and SON, that contain long low complexity regions rich in arginine and serine dipeptides (RS domain) and form a dense meshwork via multivalent interactions (Ilik et al., 2020). RS domains are also a feature of many components of the splicing machinery and other RNA processing factors, including SR proteins (SRSF1-SRSF12) (Wegener and Muller-McNicoll, 2019). Through multivalent RS-RS interactions SR proteins are retained in nuclear speckles and stored in an inactive state, but during stress or changes in transcription they are activated and released to the nucleoplasm. RS-RS interactions and hence nuclear speckle residency is modulated through PTMs that control the RNA-binding and phase separation propensities of nuclear speckle RBPs and retained mRNPs (Snead and Gladfelter, 2019). Recent high-throughput proteomic screens revealed that splicing components, including SR proteins, are also prime targets of SUMOylation, and some members of the SUMOylation machinery, e.g. UBC9, also localize to nuclear speckles (Richard et al., 2017). Moreover, it was shown that SRSF1, which is involved in assembly of the A complex, promotes SUMOylation of RNA processing factors, in particular in response to heat stress through interaction with UBC9 (Pelisch et al., 2010). This led to the proposition that SUMOylation might be required for spliceosome assembly and splicing efficiency (Pozzi et al., 2018). In line with this idea, addition of a recombinant SUMO-isopeptidase decreases the efficiency of splicing in *in vitro* assays pre-mRNA splicing assays (Pozzi et al., 2017). Moreover, a SUMO-deficient variant of PRP3, a component of the U4/U6 di-snRNP, fails to co-precipitate U2 and U5 snRNAs and the splicing factors SF3 and Snu114, suggesting that SUMOylation of PRP3 promotes U4/U6U5 tri-snRNP formation (Pozzi et al., 2017; Figure 2). This PRP3 mutant also exhibited diminished recruitment to active spliceosomes and did not rescue splicing defects observed in PRP3-depleted cells. Interestingly, however, we have recently shown that the PRP19 splicing complex is tightly associated with the SUMO peptidase SENP6, suggesting that SUMO deconjugation of the spliceosome is also needed for proper splicing (Wagner et al., 2019). This idea is also supported by recent findings from the Lamond lab, which linked impaired SUMO deconjugation to the inhibition of splicing (Pawellek et al., 2017). The authors proposed that treatment of cells with the splicing inhibitor hinokiflavone, a plant-derived biflavonoid, inhibits SUMO deconjugases. They also demonstrated that hinokiflavone prevents transition of the spliceosome from the A complex to the catalytic activated B complex and proteomic studies revealed that this was accompanied by dramatically enhanced SUMOylation of U2 snRNP proteins. Their data suggest that deSUMOylation of U2 components is needed for formation of the activated B complex. Although these data provide strong circumstantial evidence for a role of conjugation-deconjugation in controlling spliceosome dynamics, it remains to be demonstrated that the lack of deSUMOylation in response to hinokiflavone is directly responsible for the observed splicing defects. SUMOylation might also affect the dynamics of nuclear speckles. Indeed, splicing inhibition by hinokiflavone changed the morphology and composition of nuclear speckles. They now formed “mega-speckles” that accumulated SUMO1/2/3, splicing factors, snRNPs and unspliced, polyadenylated mRNAs (Pawellek et al., 2017). Enlarged nuclear speckles have also been observed with other splicing inhibitors (Araki et al., 2015; Carvalho et al., 2017), but it is currently unknown whether they impair the deSUMOylation pathway.

## SUMO and the Dynamics of Stress Granules

The best-studied example of cytosolic MLOs are stress granules (SGs), which form through LLPS in response to various stress conditions, including heat or oxidative stress (Protter and Parker, 2016). SGs are ribonucleoprotein particles comprised of untranslated mRNAs and RBPs. Their assembly is tightly linked to the inhibition of translation initiation, which helps in relieving cellular protein quality control systems from additional protein influx during stress exposure. Together with mRNA, stalled translation pre-initiation complexes comprising 40S ribosomal subunits and translation initiation factors provide the seed for further recruitment of cytosolic and nuclear RBPs, such as G3BP, FMR1, FUS, or TDP-43. The mRNAs stored in SGs can be either directed toward mRNA decay or their translation can be reinitiated when stress is released and SGs disassemble. The mechanism of SG formation and dissolution are still not fully understood, but there is accumulating evidence that post-translational modifications contribute to these processes (Turakhiya et al., 2018; Hofweber and Dormann, 2019; Hofmann et al., 2021; Tolay and Buchberger, 2021). Recent independent findings by the Hornstein and Müller groups suggest that the SUMO system and the StUbL pathway are critically involved in both assembly and dissolution of SGs (Keiten-Schmitz et al., 2020; Marmor-Kollet et al., 2020). A role of SUMOylation in modulating the formation and composition of SGs was initially inferred from work on eIF4A2, a subunit of the cap-binding eIF4 complex (Jongjitwimol et al., 2016). Watts and co-workers reported that recruitment of eIF4A2 to SGs upon arsenite-induced oxidative stress goes along with its enhanced SUMOylation, whereas expression of a SUMOylation deficient mutant of eIF4A2 results in impaired SG formation. Work by the Hornstein laboratory now supports the idea that SG assembly or targeting may involve SUMOylation (Marmor-Kollet et al., 2020). It was observed that mutation of two reported SUMOylation sites in FMR1 leads to its reduced recruitment to SGs in response to arsenite. Furthermore, delayed SG formation in response to arsenite was detected upon inhibition of the SUMO E2 enzyme UBC9 prior to stress exposure, by genetic means or by small molecule inhibitors, suggesting that SUMOylation of SG-associated proteins is involved in their recruitment to these structures. These findings are consistent with mass-spectrometry-based SUMO proteomics that identified many SG-associated RBPs as stress-induced SUMOylation targets and APEX-based proximity-proteomics that detected SUMO at SGs (Matic et al., 2009; Hendriks and Vertegaal, 2016; Marmor-Kollet et al., 2020). However, endogenous SUMO has so far never been stably detected within SGs by immunofluorescence, thus it remains unclear whether SUMO functions as an essential glue-like scaffold in SGs. In an alternative model transient SUMO conjugation may prime SG components for recruitment and assembly in SGs. Once incorporated into the complex SUMO could be removed potentially explaining why only a small fraction of a substrate is modified at a given time (Hay, 2005). For validation of this model, it remains to be determined where SUMO conjugation and deconjugation of SG components occurs. Since SUMO ligases (e.g., RanBP2) and isopeptidases (SENP1 and SENP2) are associated with nuclear pore complexes, transient SUMOylation of nuclear RBPs may occur upon nucleo-cytoplasmic shuttling (Flotho and Melchior, 2013; Cappadocia and Lima, 2018; Kunz et al., 2018).

While the above-mentioned data involve SUMO in SG targeting and assembly, the SUMO pathway is also critical for SG disassembly upon stress release. In a search for stress-induced targets of RNF4 we identified and validated a large number of SG-associated RBPs, including the nuclear RBPs FUS and TDP-43, as targets of SUMO-primed ubiquitylation (Keiten-Schmitz et al., 2020). We further found that impairment of the StUbL pathway by chemical or genetic inhibition of SUMO2/3 or depletion of RNF4 significantly delays SG clearance in cells recovering from heat or arsenite-induced proteotoxic stress. By contrast, overexpression of the chain-selective SUMO isopeptidases SENP6 or SENP7 triggers SG assembly. Altogether, these data show that SUMOylation and polySUMO-primed ubiquitylation by RNF4 fosters the disassembly of SGs. This concept was strengthened by work from Hornstein and co-workers (Marmor-Kollet et al., 2020). Marmor-Kollet et al. used APEX-based proximity-proteomics to characterize the SG-associated proteome in response to stress induction and release. Among a set of “disassembly engaged proteins,” which are specifically associated with SG proteins when they disassemble, they identified and validated the SUMO E1 subunit AOS1 (alias SAE1), the E2 UBC9, and the SUMO E3 ligases TOPORS and RANBP2. It was further demonstrated that inhibition of SUMOylation by siRNA-mediated depletion of AOS1 or UBC9, or small molecule inhibitor of UBC9 (2D08), impaired SG disassembly. The ensemble of these data provides compelling evidence that SUMOylation is functionally connected to SG disassembly. However, important mechanistic questions are still open. For example, it remains to be determined whether SUMOylation and RNF4-mediated ubiquitylation occur directly on disassembling SGs or at a later stage, for example when nuclear SG-associated proteins re-enter the nucleus. Since we were unable to detect RNF4 at SGs and found stress-induced SUMO conjugates predominantly compartmentalized in the nucleus, we favor a model of RNF4-mediated ubiquitylation taking place in the nuclear compartment at PML NBs. In support of this, we observed that lack of PML also impairs SG disassembly. Based on these data we propose that in response to proteotoxic stress the StUbL pathway primarily targets the nuclear fraction of SG-associated RBPs thereby bridging nuclear to cytosolic protein quality control. To reconcile this concept with data from Marmor-Kollet et al. one possible scenario might be that SUMO-priming occurs at SGs upon their disassembly, whereas subsequent polySUMOylation and ubiquitylation primarily involves the nuclear StUbL machinery. Regardless of these molecular details, a compelling hypothesis is that—similar to what is described for PML NBs—the SUMO system controls both the assembly and dissolution of SGs. Whether this dual function is also controlled by a switch from mono- to polySUMOylation needs to be addressed in future experiments.

Importantly, these data also open up new perspectives in the understanding of neurodegenerative disease, such as amyotrophic lateral sclerosis (ALS) or frontotemporal lobar degeneration (FTLD) which have been linked to aberrant and persistent SGs (Wolozin and Ivanov, 2019). In a subset of ALS or FTLD patients, mutations in FUS or TDP-43 induce a transition of SGs from a liquid-like dynamic to a solid state and FUS/TDP-43 aggregates are found in affected brain regions of patients suffering from ALS or FTLD. Interestingly, we could demonstrate that the StUbL pathway limits the formation of aberrant SGs caused by expression of the ALS-associated FUS^*P*525L^ mutant, pointing to a possible role of SUMO in protecting from ALS pathology (Keiten-Schmitz et al., 2020; Marmor-Kollet et al., 2020). In support of this idea, Marmor-Kollet and colleagues provided evidence that impairment of the SUMO pathway may affect formation of aberrant SGs and ALS pathology in the context of *C9orf72* mutations. Genetic alterations of the *C9orf72* gene, due to expansion of a GGGGCC hexanucleotide repeat in the first intron, represent the most frequently observed inherited form of ALS and generate different dipeptide repeat proteins. Intriguingly, expression of one of these dipeptides, the poly-PR(50) repeat protein, impaired SUMO ligase recruitment to SGs and SG SUMOylation. Further, enhanced SUMOylation activity ameliorated photoreceptor neurodegeneration in a *drosophila* model of *C9orf72*-related ALS (Marmor-Kollet et al., 2020). How expression of poly-PR(50) dipeptide repeat proteins inhibits SUMOylation activity at SGs is currently unknown. Notably, poly-PR(50) is found in nuclear aggregates indicating that it might sequester the SUMO machinery in these aggregates.

## Conclusion and Perspectives

Investigation of MLOs and characterization of their assembly-disassembly mechanisms are an emerging field of biophysics and cell biology. The role of SUMO in controlling MLO dynamics likely goes beyond the above-mentioned examples, since formation of Cajal bodies (alias coiled bodies), processing bodies (P-bodies, PBs) and the recently described NELF bodies are also controlled by SUMOylation. Thus, SUMOylation and a SIM-like-domain in SMN are critical for the assembly of Cajal bodies thereby likely controlling in snRNP and snoRNP biogenesis (Tapia et al., 2014). P-bodies are cytoplasmic RNPs with functions in translational repression and/or mRNA decay. PBs and SGs share a close relationship and exchange RNAs as well as proteins. One example is the RNA helicase DDX6, which was shown to be associated with SUMO E3 ligase TIF1β and a number of SUMOylation substrates (Bish et al., 2015). A very recent example in SUMO-dependent phase separation is the formation of heat-induced NELF (negative elongation factor)-containing condensates (Rawat et al., 2021). The NELF complex is a hetero-tetramer composed of the subunits NELFA, B, C/D, and E. In response to heat stress NELF forms nuclear condensates that drive transcriptional downregulation and cellular survival under stressful conditions. It has been proposed that these structures represent nuclear counterparts of cytosolic stress granules functioning as critical nodes of cellular stress survival by adapting gene expression programs. NELF condensates cause transcriptional pausing by negatively regulating transcriptional elongation by RNA polymerase II. Intriguingly, stress-induced SUMOylation and the E3 SUMO ligase ZNF451 are required for NELF condensation providing another intriguing example how the SUMO system integrates the cellular stress response with phase separation. Another important aspect for future research concerns the role of SUMO conjugation-deconjugation in regulating the interdependency and interplay of distinct MLO, such as PML NBs with nucleoli and SGs, under stress (Condemine et al., 2007; Keiten-Schmitz et al., 2020).

## Author Contributions

JK-S, LR, EH, MM-M, and SM wrote the article. JK-S and LR designed the figures. All authors contributed to the article and approved the submitted version.

## Conflict of Interest

The authors declare that the research was conducted in the absence of any commercial or financial relationships that could be construed as a potential conflict of interest.
